# Detection of preterm birth in electrohysterogram signals based on wavelet transform and stacked sparse autoencoder

**DOI:** 10.1371/journal.pone.0214712

**Published:** 2019-04-16

**Authors:** Lili Chen, Yaru Hao, Xue Hu

**Affiliations:** 1 School of Mechatronics & Vehicle Engineering, Chongqing Jiaotong University, Chongqing, China; 2 School of Chongqing Key Laboratory of Urban Rail Transit Vehicle System Integration and Control, Chongqing Jiaotong University, Chongqing, China; 3 Department of Blood Transfusion, The First Affiliated Hospital of Chongqing Medical University, Chongqing, China; Liverpool John Moores University, UNITED KINGDOM

## Abstract

Based on electrohysterogram, this paper designed a new method using wavelet-based nonlinear features and stacked sparse autoencoder for preterm birth detection. For each sample, three level wavelet decomposition of a time series was performed. Approximation coefficients at level 3 and detail coefficients at levels 1, 2 and 3 were extracted. Sample entropy of the detail coefficients at levels 1, 2, 3 and approximation coefficients at level 3 were computed as features. The classifier was constructed based on stacked sparse autoencoder. In addition, stacked sparse autoencoder was further compared with extreme learning machine and support vector machine in relation to their classification performance of electrohysterogram. The experiment results reveal that classifier based on stacked sparse autoencoder showed better performance than the other two classifiers with an accuracy of 90%, a sensitivity of 92%, a specificity of 88%. The results indicate that the method proposed in this paper could be effective for detecting preterm birth in electrohysterogram and the framework designed in this work presents higher discriminability than other techniques.

## Introduction

Preterm birth is defined as birth before 37 weeks of pregnancy. The World Health Organization have reported that every year about 15 million newborns are preterm, which account for more than 10% of all babies born around the world. [1] Preterm birth is a dominant cause for morbidity and mortality during both the perinatal and early neonatal periods. In addition, the complications of preterm birth such as significant neurological, mental, behavioral and pulmonary problems have a significant adverse impact on family and the economy.[2] Preterm birth is therefore both a major medical and economic challenge. One of the keys to reduce the incidence of preterm birth would be its detection or prediction using effective methods.

For the detection or prediction of preterm birth, continuous efforts have been applied toward diagnosing the onset of early labor for many years. As a result, various clinical techniques have been designed to monitor labor, such as the index detection based on biochemical indicators, infection immunity index and biophysical indicators. To prevent spontaneous preterm birth within asymptomatic high-risk women, Crane and Hutchens [3] adopted transvaginal ultrasonography to investigate the ability of cervical length. In order to predict the onset of preterm birth, Hudić et al. [4] studied the maternal serum concentration of progesterone-induced blocking factor. Compared with those methods, recording and analyzing EHG signals is a new noninvasive technique for diagnosing preterm birth, which has been proved to be of interest to provide a better monitor for the process of pregnancy. [5, 6].

In conjunction with EHG signals processing, extracting features from EHGs is an important step which can catch uterine contractility information. Among the EHGs analysis approaches, linear methods in both time and frequency domains were first adopted to extract features. Maner et al. [7] studied the ability of the power density spectrum peak frequency to classify EHG signals recorded on pregnant women who were 48, 24, 12 and 8 hours from term delivery, and 6, 4, 2 and 1 day(s) from preterm delivery. Some time-frequency analysis methods were also adopted to characterize EHG signals. Arora et al. [8] extracted the relative wavelet energy from each detail based on 4-level decomposition. Hassan et al. [9] employed wavelet coherence to diagnose the uterine electrical activity synchronization in labor. In an attempt to better characterize EHG, nonlinear characteristics have been explored to analyze EHG signals. Radomski et al. [10] adopted sample entropy to assess regularity in EHGs. Diab et al. [11] explored the performance of four nonlinear methods (time reversibility, sample entropy, Lyapunov exponent and delay vector variance) for detecting preterm birth. It is known that uterus is composed of a huge amount of intricately interconnected cells, which lead to uterus a complex, non-stationary and non-linear dynamic system. [12] Therefore, time-frequency analysis methods combined with non-linear signal processing techniques may achieve better results in analyzing EHG signals. Within time-frequency analysis methods and non-linear signal processing techniques, wavelets can capture the subtle changes in transient signals and entropy [13–15].

To be successful in classification and recognition for EHG signals, selection of a reliable classification method should also be a careful consideration. Recently, many recognition algorithms including artificial neural network, decision trees, Bayesian classifier, support vector machine, extreme learning machine and deep learning have been proposed for pattern recognition. Maner and Garfield [2] adopted an artificial neural network on EHG data to distinguish human term and preterm labor. In this work, a classification accuracy of 80% was achieved. Lu et al. [16] used multilayer Perceptron to distinguish EHG signals between 11 preterm signals and 28 term signals. This work reported that the classification accuracy of 64.1%. Moslem et al. [17] used a competitive neural network to classify EHG signals recorded on 32 women as pregnancy group and labor group. As a result, a classification accuracy of 78.2% was achieved. Moslem et al. [18] applied support vector machine to determine 137 pregnancy contraction signals and 76 labor signals. This work obtained an overall classification accuracy of 83.4%. While among these recognition technologies, most of them are supervised learning which needs millions of labeled samples for training. [19] In addition, the neural network can easily get into local optima, thus leading to a poor generalization. So, the choice of recognition model still is a big challenge because of the complex classification problem. A new unsupervised feature learning method, sparse autoencoder (SAE) possesses strong representation ability which can reveals high-level representation from complex data. Stacked sparse autoencoder (SSAE), a kind of deep learning neural network consisted of multiple layers of SAE, has achieved remarkable results in many fields. [20–24].

**Fig 1:**
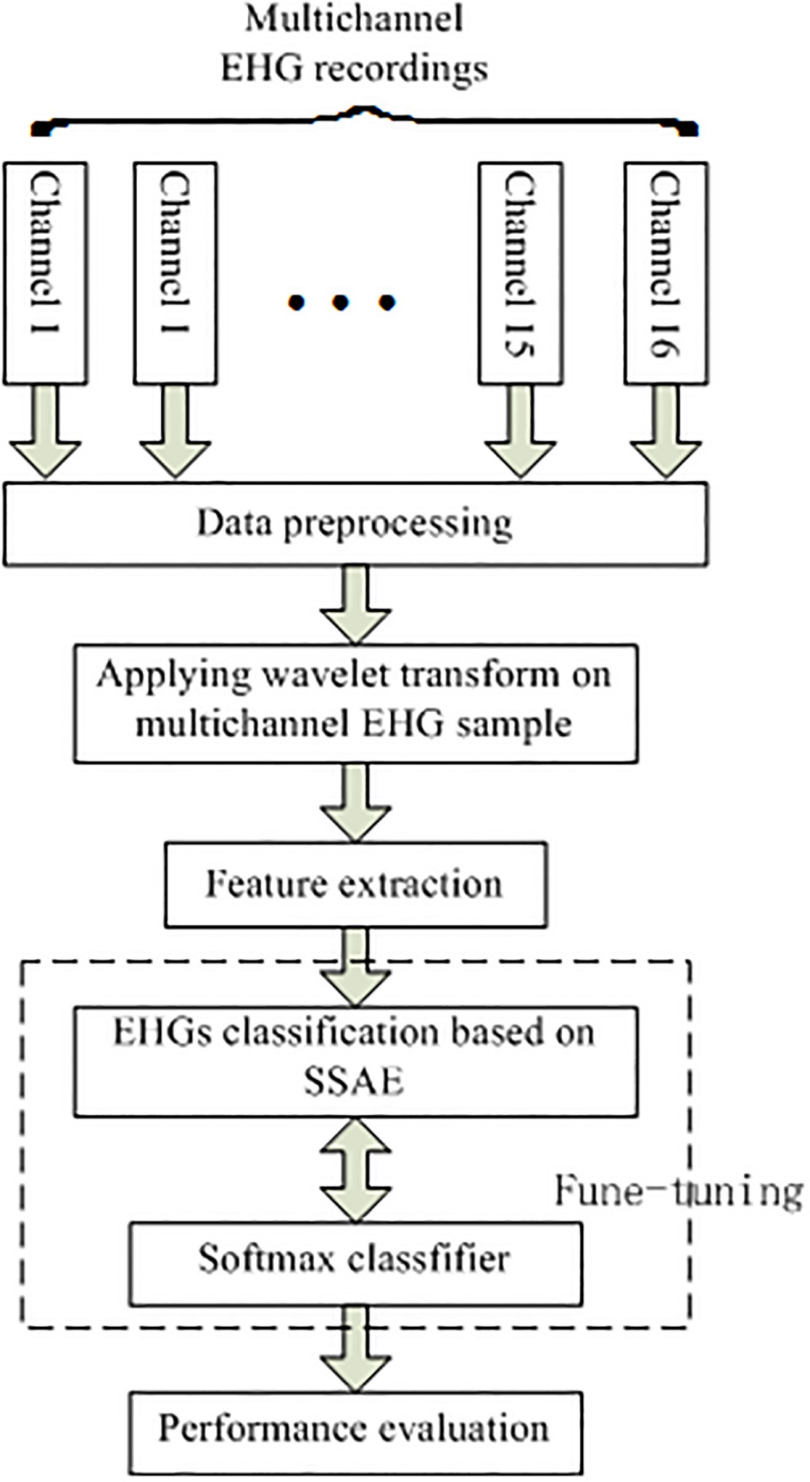
The generalized block diagram of the proposed method.

In present work, this paper proposed a novel method for classifying pregnancy EHG signals and labor EHG signals based on wavelet transform, sample entropy and stacked sparse autoencoder. The rest of this paper is composed as follows. After the introduction, the dataset used in this work and the methods of wavelet transform, sample entropy and stacked sparse autoencoder are described in Section 2. The process of the experiment and the obtained experiment results are discussed in Section 3. Section 4 concludes this paper. The block diagram of the designed method is presented in Fig 1.

## Materials and methods

### Data description

The EHG recordings employed in this work derives from Icelandic 16-electrode Electrohysterogram Database of PhysioNet. [25, 26] This dataset comprises 112 pregnancy EHG recordings and 10 labor EHG recordings. All the 122 EHG recordings were taken from 45 participants using a 4-by-4 electrode matrix positioned on the women’s abdomen. Pregnancy recordings were performed on participants who are in the third trimester and not suspected to be in labor. This experiment was made at Akureyri Primary Health Care Centre and Landspitali University Hospital. Labor recordings were recorded on participants who are suspected to be in labor within 24 hours. The duration of pregnancy recordings was between 19–86 minutes and the duration of labor recordings were 8–64 minutes. The signal sampling rate was 200 Hz. The recording device adopted an anti-aliasing filter whose cut-off frequency was 100 Hz. The tocographic signals were obtained at the same time during recordings based on a tocodynamometer attached to women’s abdomen. The protocol was approved by the National Bioethics Committee in Iceland (VSN 02-006-V4).

### Wavelet transform and entropy calculation

Wavelet transform, a powerful signal processing technique, was first proposed to overcome the shortcomings of Fourier transform. Wavelet transform is a multi-revolution decomposition fast algorithm which can decompose a time series into a set of components by utilizing orthogonal wavelet bases. [27] It is equal to recursively decomposing the time series using a high-pass and low-pass filter pair. The band width of high-pass filter is equal to that for low-pass filter. Therefore after each level of decomposition, the sampling frequency is halved. The low-frequency component then be recursively decomposed to produce the components of the next stage. [28].

Given *ψ* ∈ *L*^2^(*R*) is a mother wavelet satisfying the permission condition ∫_*R*_ *ψ*(*t*)*dt* = 0. The wavelet cluster can be expressed as:

$$\psi_{s,c}(t)=\frac{1}{\sqrt{s}}\psi \left(\frac{t-c}{s}\right),s>0,c\in R$$ (1)

In formula (1), *s* is the scale parameter and *c* is the translation parameter.

Then continuous wavelet transforms *W*(*s*, *c*) of *x*(*t*) can be expressed as:

$$W\left(s,c\right)=\frac{1}{\sqrt{s}}\int x\left(t\right)\psi^{*}\left(\frac{t-c}{s}\right)dt$$ (2)

In formula (2), * indicates complex conjugation.

Then discrete wavelet transform of time series *x*(*n*) (*n* = 1, …, *N*) can be obtained based on continuous wavelet transform by restraining *s* and *c* to a discrete lattice (*s* = 2^−*j*^, *c* = 2^−*j*^ *k*):

$$\left\{ \begin{array}{l} cA_{j,k}(n)=\left[\sum_{n}x(n)l_{j}^{*}(n-2^{j}k)\right]\\ cD_{j,k}(n)=\left[\sum_{n}x(n)h_{j}^{*}(n-2^{j}k)\right] \end{array}\right.$$ (3)

In formula (3), *j* refers to the wavelet scale and *k* refers to translation factors. *l*(*n*) denotes low pass filter and *h*(*n*) denotes high pass filter. *cA*_*j*,*k*_ and *cD*_*j*,*k*_ represent the coefficients of approximation components and detail components respectively.

Then the frequency band ranges contained in *D*_*j*_(*k*) and *A*_*j*_(*k*) can be achieved by reconstruction as follows.

$$\left\{ \begin{array}{l} D_{j}(k):\left[2^{-(j+1)}f_{s},2^{-j}f_{s}\right]\\ A_{j}(k):\left[0,2^{-(j+1)}f_{s}\right] \end{array}\right.(j=1,2,\ldots ,J)$$ (4)

In formula (4), *f*_*s*_ is the sampling frequency and *J* refers to maximal scale.

The time series *x*(*n*) can be denoted by the sum of all components as follows.

$$x(n)=D_{1}(n)+A_{1}(n)=D_{1}(n)+D_{2}(n)+A_{2}(n)=\sum_{j=1}^{J}D_{j}(n)+A_{J}(n)$$ (5)

In this work, the sample entropy will be used for its effective calculation power in complexity. Detailed descriptions for sample entropy method can be found in Ref[29]. The definition of sample entropy is as follows.

$$\text{SampEn}\left(m,r,N\right)=-\text{ln}\left[\frac{B_{}^{m+1}\left(r\right)}{B_{}^{m}\left(r\right)}\right]$$ (6)

In formula (6), *m* is the embedding dimension and *r* is vector comparison threshold. *B*^*m*^ (*r*) stands for the probability of two sequences matching for *m* points, and *B*^*m*+1^ (*r*) is the probability of two sequences matching for *m* + 1 points.

### Sparse autoencoer

**Fig 2:**
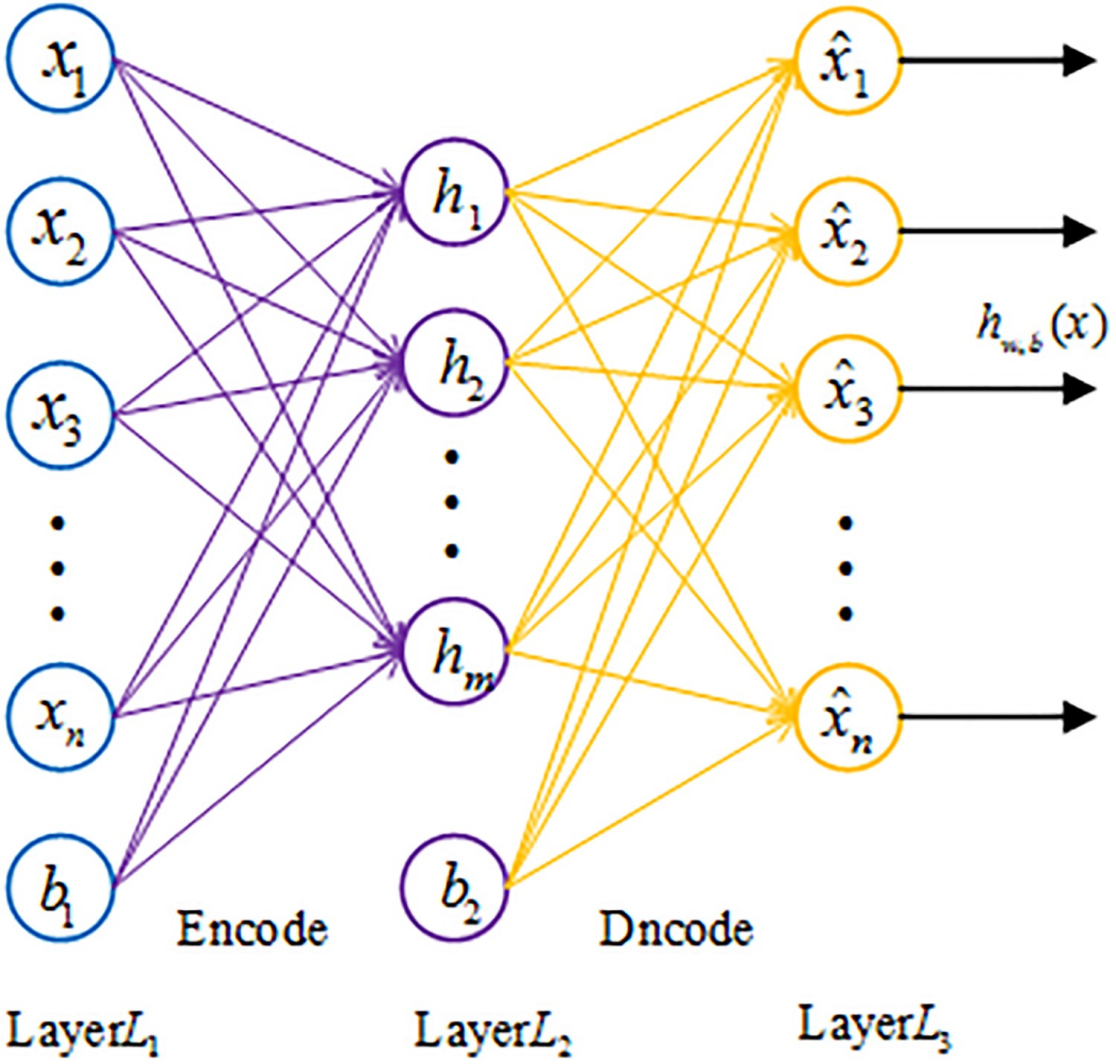
The structure of an autoencoder.

As an unsupervised learning method, the autoencoder is a multi-layer feedforward neural network used to represent the input with backpropagation. The autoencoder aims to learn a high-level representation from input data with minimum reconstruction loss. The basic structure of a three-layer autoencoder is described in Fig 2. A sparse autoencoder is an extension of the autoencoder which can achieve some specific representation ability by introducing a spare constraint on the hidden layer. [30].

The algorithm steps for the sparse autoencoder are given as follows.

Step 1: Given dataset *X* = {*x*(1), *x*(2), …, *x*(*i*), …, *x*(*N*)}, *x*(*i*) ∈ *R*^*M*^ was first mapped to the hidden layer by means of a nonlinear activation function (formula (7)):
  $$Z=f(W_{1}X+b_{1})=\frac{1}{1+\text{exp}[-(W_{1}X+b_{1})]}$$ (7)
  In formula (7), the resulting hidden representation *Z* is then mapped back to a reconstructed vector *h*_*w*,*b*_(*x*) in the input space and *f* represents the encoder activation function. In formula (7), *W*_1_ and *b*_1_ represent respectively the weight matrix and the bias of the encoder.
  In the decoding process, the hidden representation *Z* is converted back to reconstructed vector *h*_*w*,*b*_(*x*) by the activation function between the hidden and output layer. The specific calculation details are as formula (8):
  $$h_{w,b}(x)=f(W_{2}Z+b_{2})=\frac{1}{1+\text{exp}[-(W_{2}Z+b_{2})]}$$ (8)
  In formula (8), *f* indicates the decoder activation function. *W*_2_ and *b*_2_ signify the weight matrix and the bias of the decoder, respectively. In formula (7) and (8), *N* is the number of data samples; *M* is the length of each data sample.
Step 2: In order to reconstruct the input data from the reconstructed vector, the parameter set {*W*_1_, *b*_1_, *W*_2_, *b*_2_} is optimized by minimizing error between the input data and the reconstructed dada. In addition, for SAE, a sparse constraint was introduced to the hidden layer. Therefore, the cost function of the SAE can be given as:
  $$J(W,b)=\frac{1}{N}\sum_{i=1}^{N}\left(\frac{1}{2}{\left\|h_{w,b}(x(i))-x(i)\right\|}^{2}\right)+\frac{\lambda }{2}\sum_{l=1}^{2}\sum_{i=1}^{s_{l}}\sum_{j=1}^{s_{l+1}}{\left(W_{ji}^{l}\right)}^{2}+\beta \sum_{j=1}^{s_{2}}KL(\rho ||{\hat{\rho }}_{j})$$ (9)
  The meanings of the notations in formula (9) are shown in Table 1.
  $KL(\rho ||{\hat{\rho }}_{j})$ can be defined as:
  $$KL(\rho ||{\hat{\rho }}_{j})=\rho \text{log}\frac{\rho }{{\hat{\rho }}_{j}}+(1-\rho )\text{log}\frac{1-\rho }{1-{\hat{\rho }}_{j}}$$ (10)
Step 3: In the process of coding, the optimal parameters of *W* and *b* need to be updated, which can be realized by minimizing *J*(*W*, *b*). This can be solved by adopting the backpropagation algorithm, where the stochastic gradient descent approach is adopted for training. The parameters *W* and *b* in each iteration can be updated as follows:
  $$W_{ji}^{l}=W_{ji}^{l}-\varepsilon \frac{\partial }{\partial W_{ji}^{l}}J(W,b)$$ (11)
  $$b^{l}=b_{}^{l}-\varepsilon \frac{\partial }{\partial b_{}^{l}}J(W,b)$$ (12)
  In formula (11) and (12), *l* represents the l^th^ layer of the network; the parameter *ε* indicates the learning rate; *i* and *j* denote the i^th^ and j^th^ neurons of two neighboring layers, respectively.

**Table 1:** The notations utilized in the cost function.

Notation	Meaning
*N*	Number of data samples
*λ*	Weight decay parameter
*β*	The weight of sparsity penalty term
*ρ*	Sparsity parameter defining the sparsity level
${\hat{\rho }}_{i}$	The average activation of hidden neuron *j*
*s*_*l*_	Number of neurons in layer *l*
*x*(*i*)	Input feature vector
*h*_*w*,*b*_(*x*(*i*))	Output feature vector
$KL(\rho ||{\hat{\rho }}_{i})$	Kullback-Leibler divergence between *ρ* and ${\hat{\rho }}_{j}$
$W_{ji}^{l}$	The weight on the connection between neuron *j* in layer *l* + 1 and neuron *i* in layer *l*

In general, the forward pass works to calculate the average activation ${\hat{\rho }}_{j}$ to get the sparse error, then the parameters were updated by using the back-propagation algorithm. After that, the effective high level representations can be obtained by the SAE[19].

### Stacked sparse autoencoder

**Fig 3:**
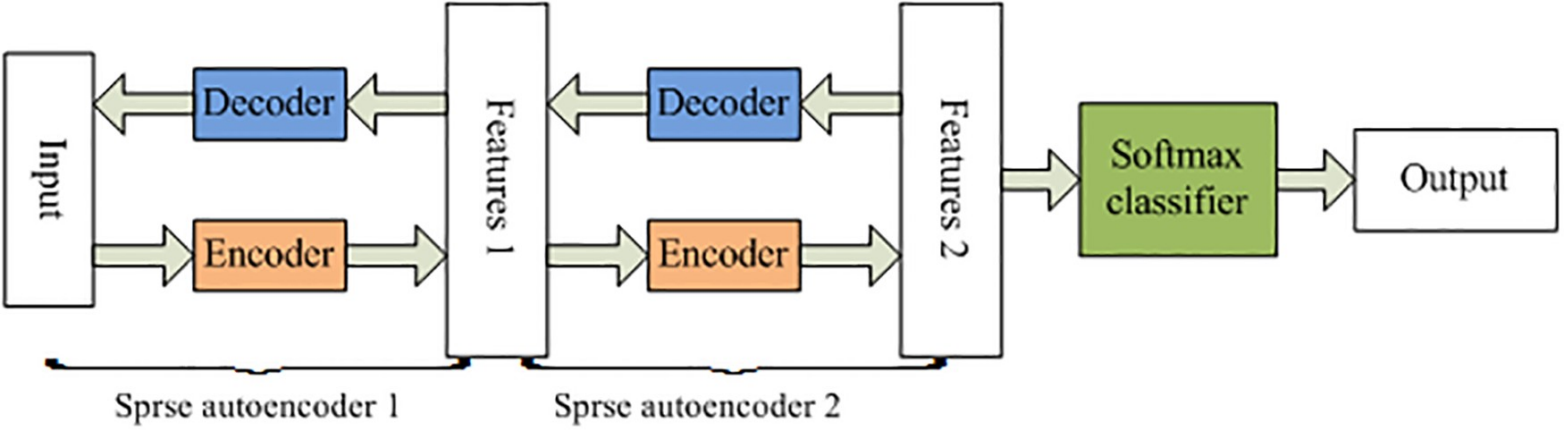
The SSAE used in this work with a softmax clssifisr.

SSAE is a deep neural network comprised of multiple layers of SAE, which adopts unsupervised greedy layer-wise algorithm to adjust the weights and uses backpropagation algorithm to fine-tune the entire deep neural networks. [31] The detailed descriptions for SSAE can be found in Ref.[32]. Fig 3 describes the SSAE structure combined with a softmax classifier.

## Results and discussion

### Data preprocessing

**Fig 4:**
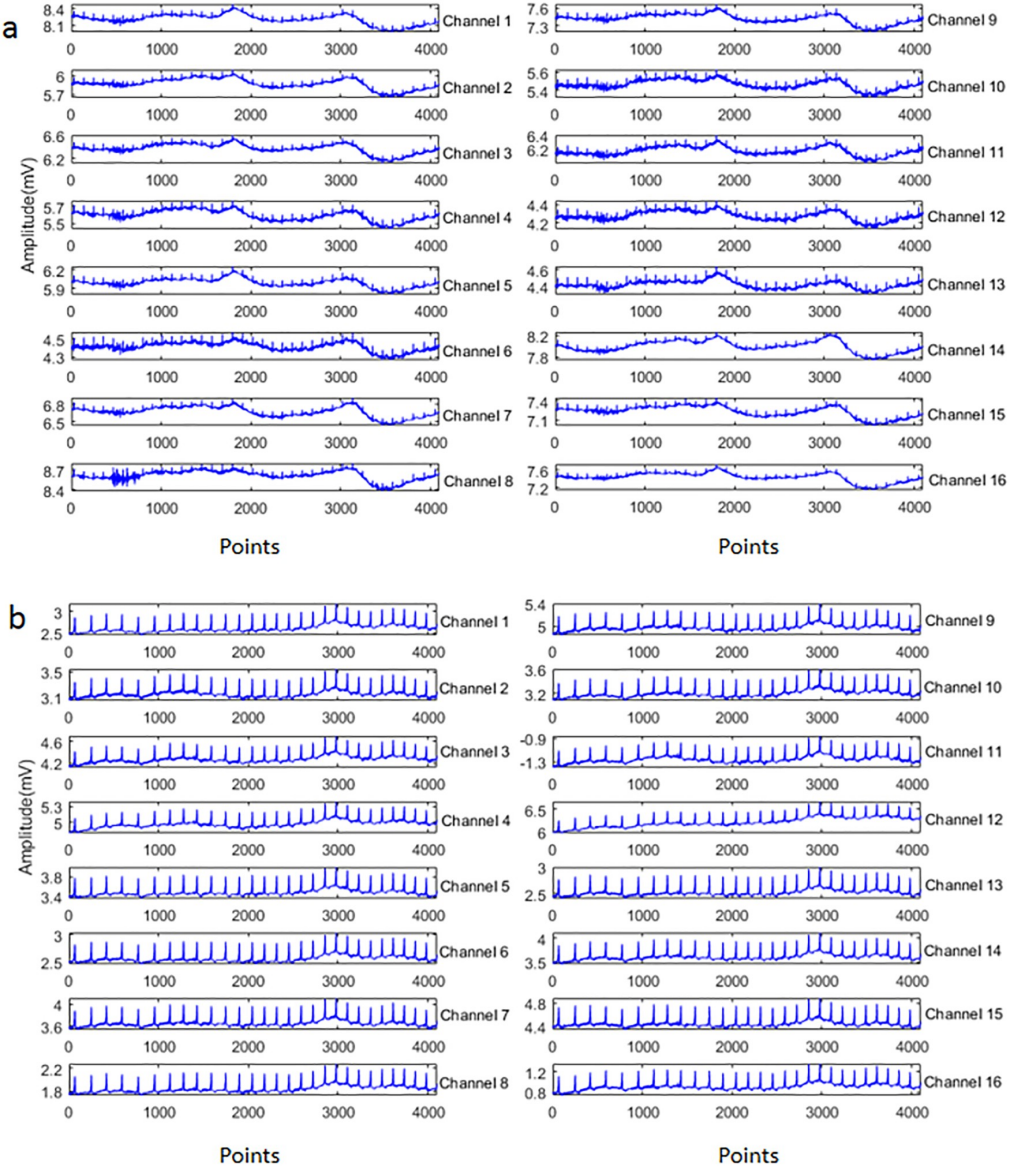
Samples of raw 16-channel EHGs. (a) Pregnancy EHG instance (b) Labor EHG instance.

**Fig 5:**
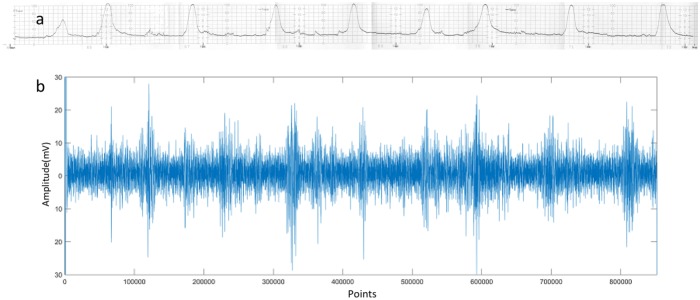
The tocographic signal and its corresponding preprocessed one time series. (a) The tocographic signal obtained by tocographic measurement (Each small square is 30 seconds). (b) The simultaneously recorded one channel when filtered between 0.1-3Hz.

It is known that the of EHG ranges between 0.1 and 3Hz. 3Hz are the most useful frequency as the other. The obtained EHG recordings often contain irrelevant information such as maternal electrocardiogram, movement artifacts and base line. [33] Fig 4 presents two original EHG instances from the pregnancy state and labor state, respectively. Therefore, before implementing features extraction, the following steps should be applied on the raw EHG recordings: first, throughout this work, all raw EHG recordings were filtered between 0.1 and 3Hz using a Butterworth filter; then, the bursts reflecting uterine contractile activity were manually segmented from each recording based on tocographic signals; Finally, 150 pregnancy EHG samples and 150 labor EHG samples were segmented from these bursts. In this study, all the 16 time series in the EHG recordings were used. The length of each time series in each sample is 4096 points. Fig 5 shows the tocographic signal and its corresponding preprocessed one time series.

### Features extraction

**Fig 6:**
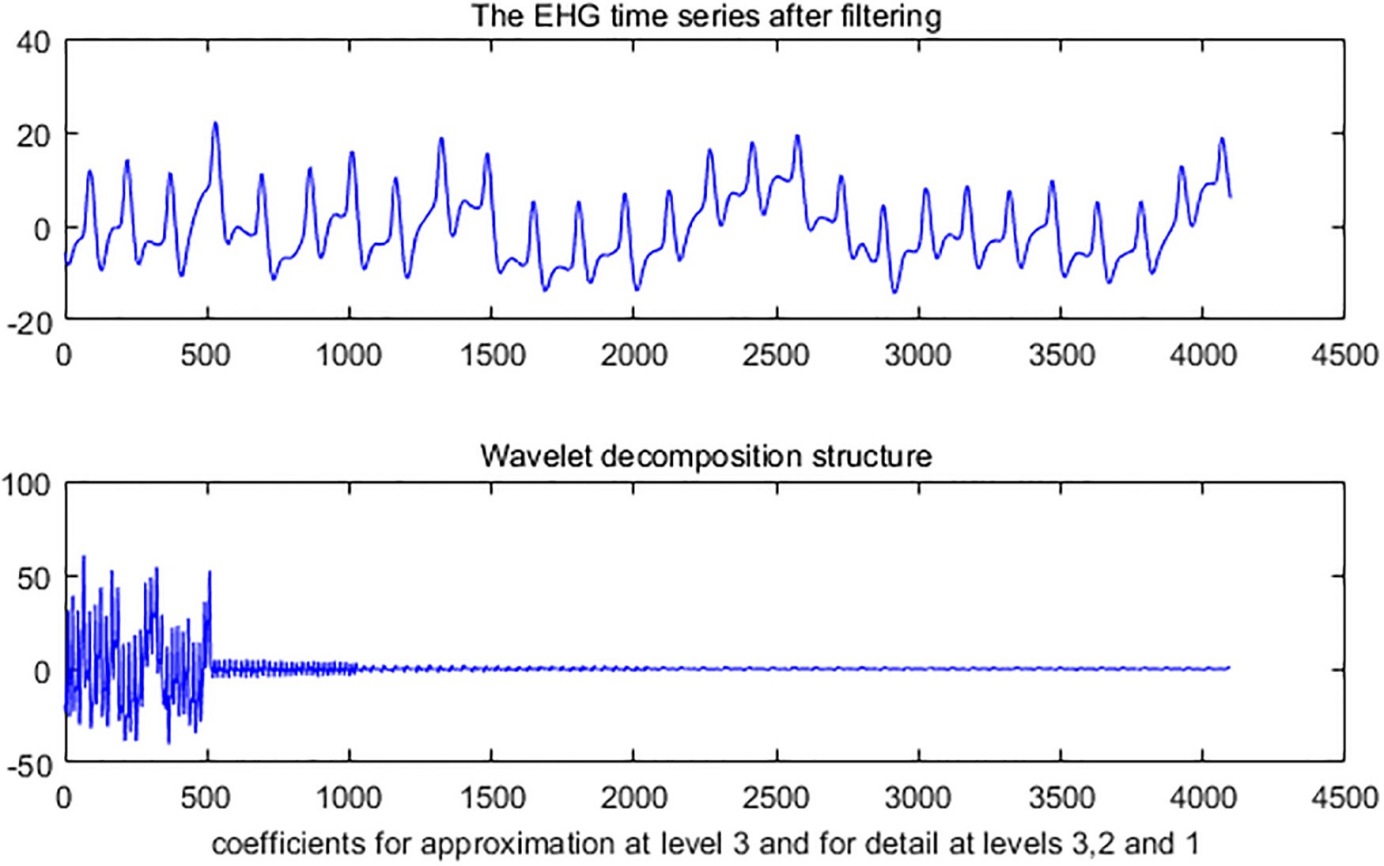
The decomposition of one EHG time series after filtering.

**Fig 7:**
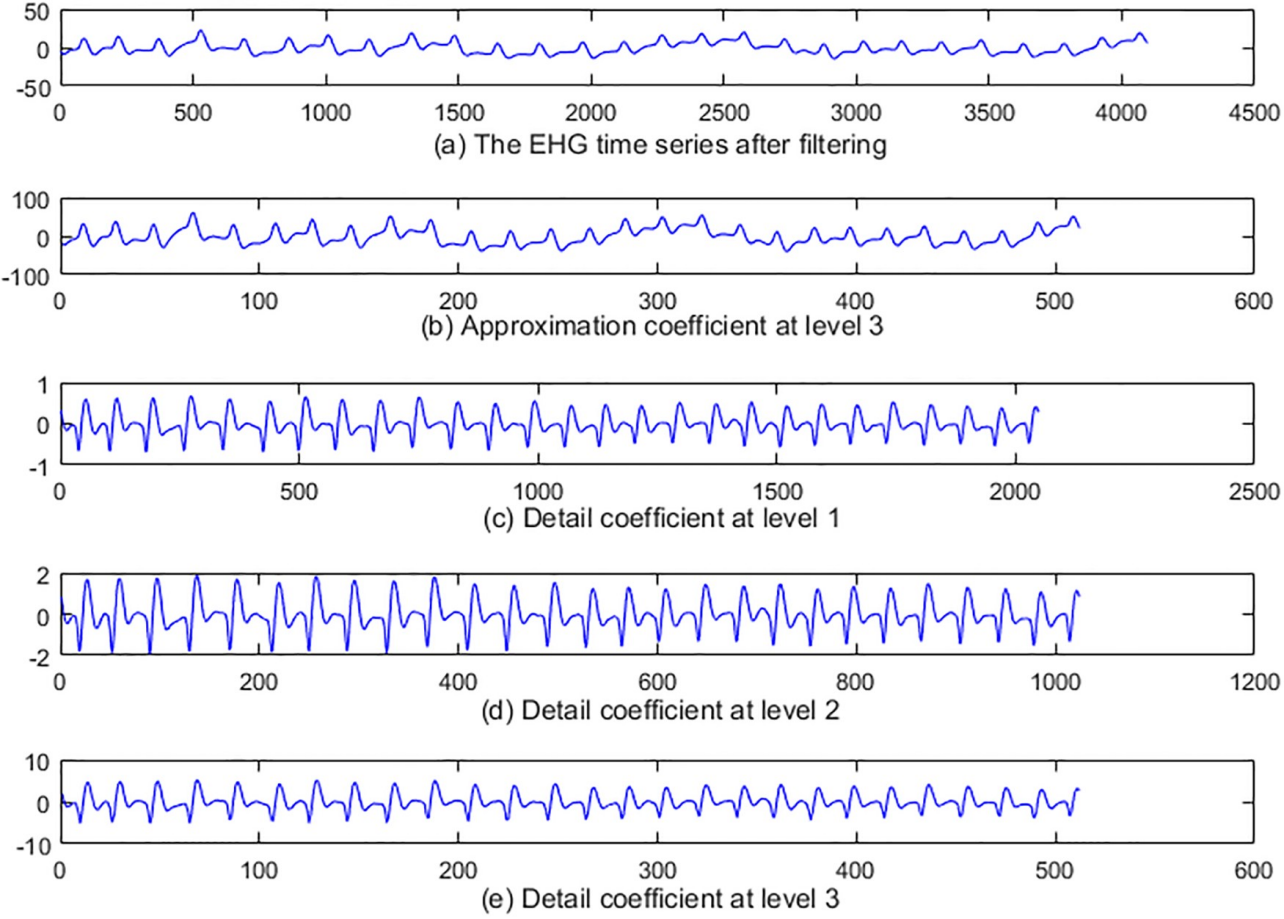
Approximate signal at level 3and detail signals at level 1, 2 and 3.

The steps for parameters extraction are as follows: Firstly, 3-level wavelet decomposition of a time series using db1 was performed. Fig 6 illustrates the result of 3-level wavelet decomposition about a time series. Secondly, the level-3 approximation coefficients of a time series are computed. Fig 7(b) shows level-3 approximation coefficients. Detail coefficients at levels 1, 2 and 3 from wavelet decomposition structure of a time series are then computed. Fig 7(c), 7(d) and 7(e) respectively illustrates the detail coefficients at levels 1, 2 and 3. Finally, Sample entropy of the detail coefficients at levels 1, 2 and 3 are then computed. Sample entropy of level-3 approximation coefficients is also computed. Thus, a feature vector consists of 64 parameters.

### SSAE classification results

**Fig 8:**
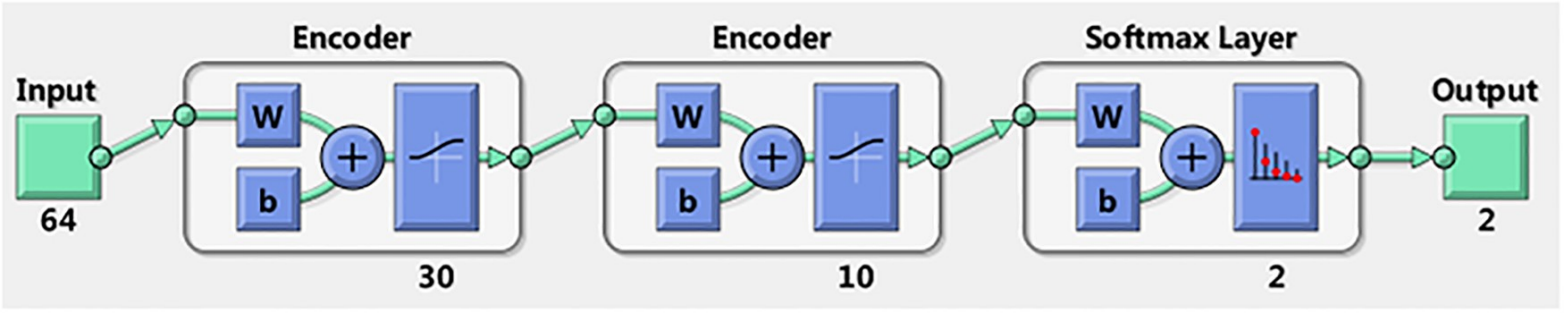
The architecture of the classification model used in this work.

**Fig 9:**
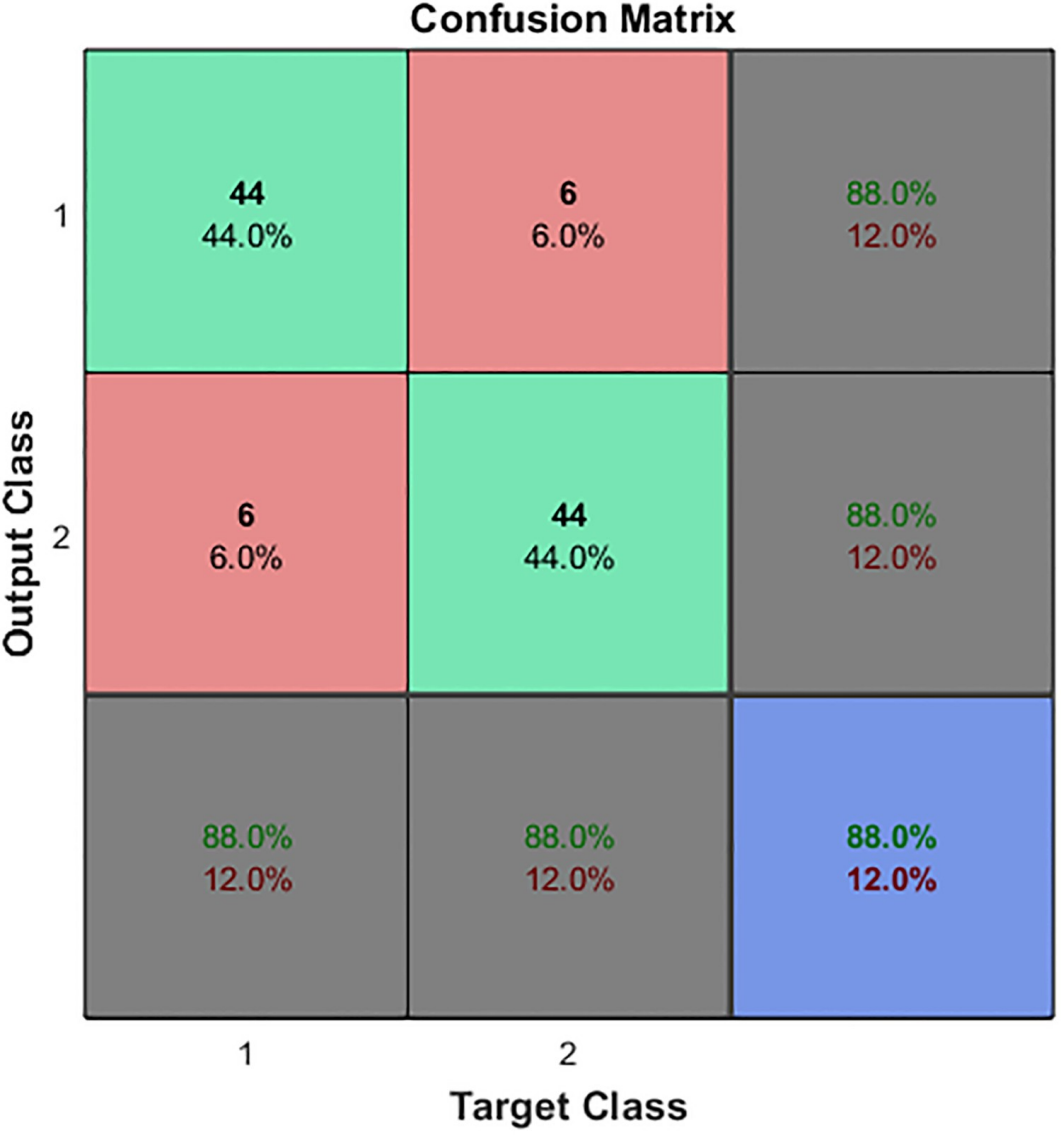
The classification result of test set by using the obtained model.

In this work, a 2-hidden-layer SSAE with a softmax classifier network is constructed to automatically classify EHG signals. As mentioned previously, 150 EHG feature vectors for pregnancy EHG and 150 EHG feature vectors for labor EHG were collected. For each vector set, 100 feature vectors were randomly chosen for training and the remaining 50 feature vectors were used for testing. When training set and test set are built, the values are all normalized to between 0 and 1. The parameter settings for this 2-hidden-layer SSAE with a softmax classifier network are given in Table 2. The architecture of the classification model used in this work is shown in Fig 8. When the classification model was established, the test set was used to verify the identification performance of the model. The confusion matrix for the proposed method in this work is presented in Fig 9. In Fig 9, the rows related to the predicted class (Output Class) and the columns related to the true class (Target Class). The first two diagonal cells indicate the number and percentage of correct classifications by the trained network. As out of 50 pregnancy cases, 44 cases are correctly classified as pregnancy, which corresponds to 88.0% of all pregnancy cases; 6 cases are incorrectly classified as labor, which corresponds to 12.0% of all pregnancy cases. Out of 50 labor cases, 44 cases are correctly classified as labor, which corresponds to 88.0% of all labor cases; 6 cases are incorrectly classified as pregnancy, which corresponds to 12.0% of all labor cases. Overall, 88.0% of all predictions are classified correctly and 12.0% are classified incorrectly.

**Table 2:** Parameter settings for modeling process.

Layer1	The number of input layer size	64
	The number of output layer size	30
	Sparsity proportion	0.05
	Sparsity regularization for loss function	8
	weight regularization for loss function	0.001
Layer2	The number of input layer size	30
	The number of output layer size	10
	Sparsity proportion	0.05
	Sparsity regularization for loss function	8
	weight regularization for loss function	0.001
Softmax classifier	The number of input layer size	10
	The number of output layer size	2
	Sparsity proportion	0.05

### Performance comparison with ELM and SVM

In order to verify the discriminability between pregnancy and labor EHG signals in this experiment, the SSAE algorithm was further compared with two widely used classification methods, namely extreme learning machine (ELM) and support vector machine (SVM). For the ELM classifier, the number of hidden neurons was set to be 20, and the Sigmoidal function was chosen as transfer function. For the SVM classifier, radial basis function was adopted as kernal function and optimal parameters were obtained in training step for every trial.

The index sensitivity, specificity and classification accuracy are used for evaluating the classification performance of the three classifiers. In addition, the classification performance for the three classifiers can be further evaluated by receiver operating characteristic (ROC) analysis, which gives us an intuitive view of entire spectrum of sensitivity.[34] The area under ROC curve (AUC) provides an effective way of comparing the performance for the classification model. The larger the area under ROC curve, the better the discrimination capability of the classification model.

The index sensitivity, specificity and classification accuracy can be represented as:

$$\text{Sensitivity}=\frac{TP}{TP+FN}$$ (13)

$$\text{Specificity}=\frac{TN}{TN+FP}$$ (14)

$$\text{Accuracy}=\frac{TP+TN}{TP+TN+FP+FN}$$ (15)

In formula (13), (14) and (15), true positives (*TP*) denotes the number of labor samples predicted correctly; false negatives (*FN*) denotes the number of labor samples predicted incorrectly; true negatives (*TN*) denotes the number of pregnancy samples predicted correctly; false positives (*FP*) denotes the number of pregnancy samples predicted incorrectly.

**Fig 10:**
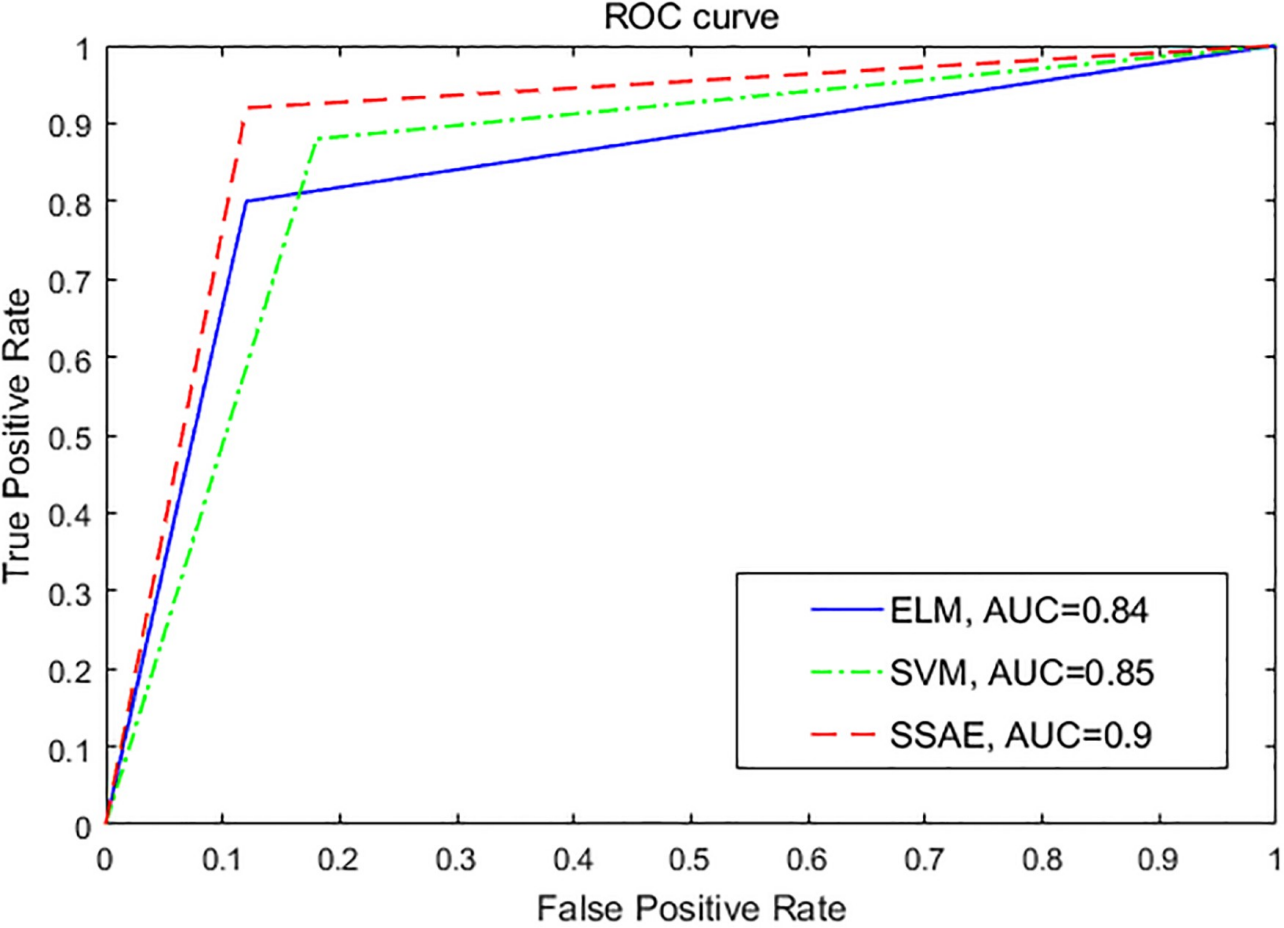
The ROC curve for the three classifiers.

**Fig 11:**
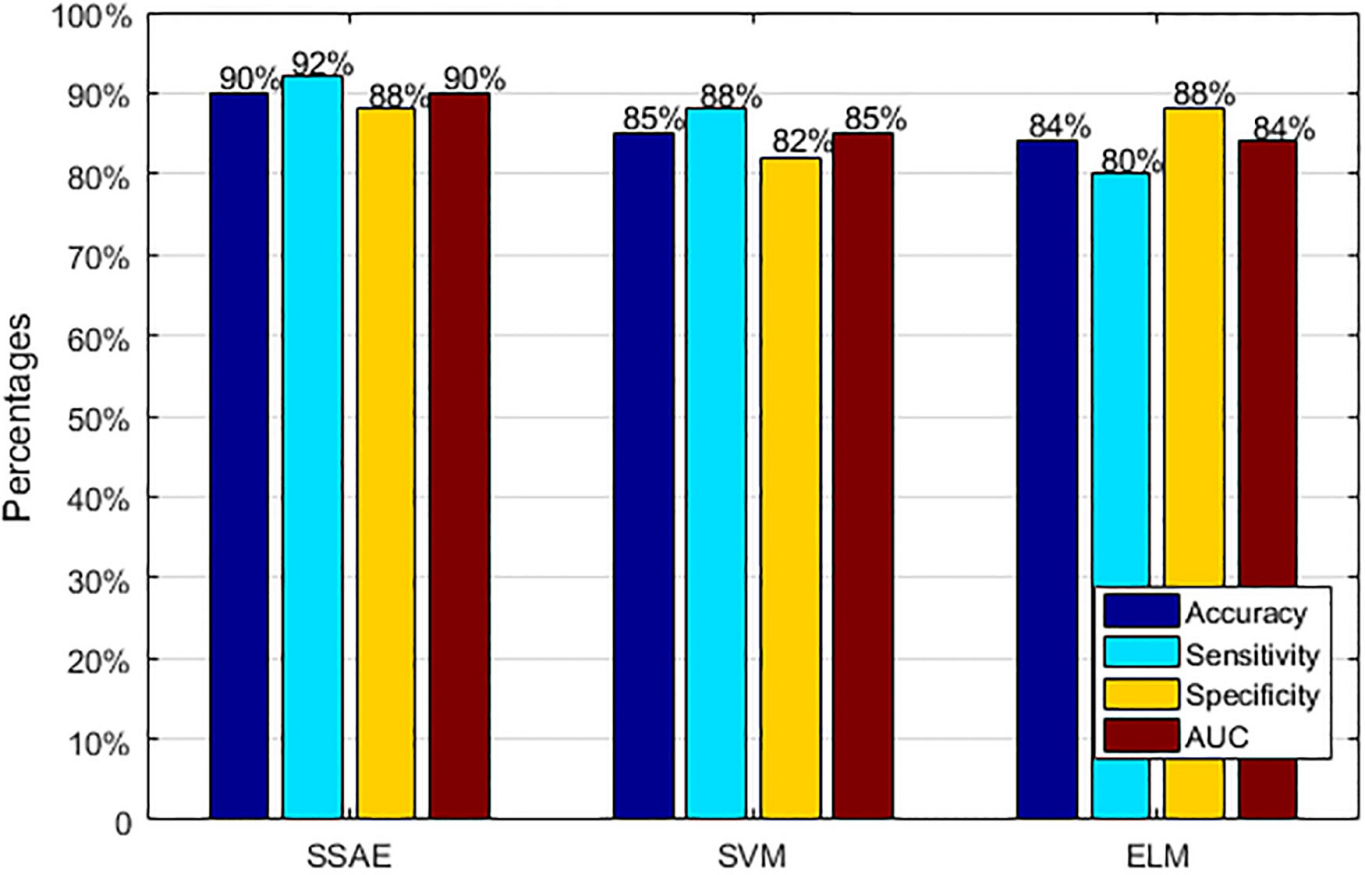
The performance comparison results for the three different classifiers using four indexes.

The performance of the three classifiers evaluated by ROC plot is illustrated in Fig 10. It is clear that the AUC achieved by SSAE is bigger than those by other two classifiers, which indicates that the classification model proposed in this work has a better classification performance. Fig 11 presents the performance comparison results for the three different classifiers using four indexes. As can be seen, when it comes to accuracy, SSAE presents the highest accuracy (90%). When it comes to sensitivity, SSAE presents the highest sensitivity (92%). When it comes to specificity, SSAE and ELM present the same sensitivity (88%). When it comes to AUC, SSAE presents the highest AUC (90%).

## Conclusion

In this work, the paper considered the application of wavelet transform and Stacked Sparse Autoencoder to the automatic classification of pregnancy EHG and labor EHG. Experimental results indicate that the method presented demonstrated better performance with an accuracy of 90%, a sensitivity of 92%, a specificity of 88% and the AUC of 90%. Experimental results demonstrated the effectiveness of feature extraction methods. It was also shown that by applying SSAE it is not only possible to improve the classification results but also to increase the generalization property of the classifier. Therefore, experimental results show that the proposed method achieves high accuracy in preterm birth detection. The method presented in this paper is a useful clinical test to aid diagnosis of preterm birth.

Further developments of this work may include that accuracy and specificity of the method is needed to be further enhanced. The classification model used in this paper has got acceptable results, but it still cannot be confidently used in clinics as accuracy of >95% is considered. In theory, as the sample increases, classifier based on SSAE will get better performance. In this paper, the experimental samples came from the database of PhysioNet with only 45 subjects. In future, more subjects will be used to train classifier based on SSAE and experiments will be performed with the new dataset in order to perfect the method in terms of specificity and accuracy.
